# Association of self-reported periodontal disease and inequities with long haul COVID-19

**DOI:** 10.1371/journal.pone.0311644

**Published:** 2024-10-10

**Authors:** Sara Alhaffar, Sriha Yalamanchi, Anubhuti Shukla

**Affiliations:** 1 Indiana University School of Dentistry, Indianapolis, IN, United States of America; 2 Department of Dental Public Health and Dental Informatics, Indiana University School of Dentistry, Indianapolis, IN, United States of America; International Medical University, MALAYSIA

## Abstract

In 2000, the Surgeon General’s report highlighted that the mouth is a mirror for overall health of an individual and that disparities in oral health are directly proportionate to general health inequities. Among patients hospitalized due to COVID-19, diabetes and cardiovascular disease are the most common comorbidities; several studies support the association of these conditions with periodontal disease. This study’s main aim is to assess the disproportionate impact of the COVID-19 pandemic on populations from lower socioeconomic statuses. The study also aims to assess the association of self-reported periodontal disease with COVID-19 disease course and severity. A sample population of Indiana residents with positive diagnosis of SARS-CoV-2 were recruited. A validated survey tool was sent to this cohort inquiring about sociodemographic distribution; co-morbid conditions, current symptoms of “long haul COVID,” course of their COVID-19 infection; history of periodontal disease, existing periodontal disease symptoms, and oral hygiene habits. 209 individuals with a history of positive COVID test were returned to the survey, and association of participant characteristics and periodontal disease-related survey items with COVID-related survey items were evaluated using chi-square tests. Lower sense of smell ratings was associated with less education (p = 0.021), being unemployed/disabled (p = 0.008), worse health status (p<0.001), more frequent bleeding gums (p = 0.031), more frequent toothache (p<0.001), lower oral health rating (p = 0.002), and vaccine status (p = 0.011). Lower sense of taste ratings were associated with older age (p = 0.018), worse health (p<0.001), more frequent bleeding gums (p<0.001), more frequent mobile or loose tooth (p = 0.010), presence of gum disease (p<0.001), more frequent loss of teeth (p = 0.013), more frequent toothache (p<0.001), worse oral health (p = 0.001), teeth lost due to gum disease (p = 0.006), and vaccine status (p = 0.001). History of hospitalization due to COVID-19 was found to be associated with a history of gum disease within the past 12 months.

## Introduction

In 2000, the Surgeon General’s Report on Oral Health clearly stated that oral health is connected to overall health and well-being [1]. The most prevalent oral diseases are dental caries and periodontal diseases, which are largely preventable. Populations at higher risk for developing medical conditions are the same as populations at higher risk for developing oral diseases [2]. Socioeconomic status refers to the absolute or relative levels of economic resources, power, and prestige closely associated with wealth of an individual, community, or country [3]. Populations of lower socioeconomic status have increased prevalence of comorbid conditions, generally poorer oral health, and more limited access to health care services, all summing up to health inequities [4]. While the COVID-19 pandemic had an impact that could be felt worldwide, populations that most experience oral health inequities disproportionately felt its effects [2]. This is due to many risk factors, a number of which were heightened by the COVID-19 pandemic: stress, alcohol use, tobacco use, poor diet, domestic violence issues, behavioral health problems, and poverty [5]. Among patients hospitalized due to COVID-19, two of the most prevalent comorbidities reported are diabetes and cardiovascular disease [5]. There is enough literature to support the association of these two conditions with periodontal disease [6, 7]. According to the Institute of Medicine and National Research Council, “poor and minority children are substantially less likely to have access to oral health care than their nonpoor and nonminority peers [4].” These populations are also more likely to lack dental insurance or depend on Medicaid. The Centers for Disease Control and Prevention (CDC) notes that “non-Hispanic Blacks, Hispanics, American Indians and Alaska Natives generally have the poorest oral health of any racial and ethnic groups in the United States,” and the same populations have been found to have significantly higher incidence of COVID-19–related infection and death [5]. A meta-analysis by Magesh et al. concluded that members of racial and ethnic minority groups had higher risks of testing positive for COVID-19 and of having a more severe disease course. They also determined that socioeconomic determinants were strongly associated with COVID-19 outcomes in racial and ethnic minority populations [8]. For example, African American and Hispanic individuals were most likely to test positive for COVID-19. Asian American individuals had the highest risk of intensive care unit admission. Decreased access to clinical care was positively associated with COVID-19 positivity in Hispanic and African American individuals [8].

Early in the COVID-19 pandemic, the American Dental Association recommended postponement of elective dental procedures, with the provision of only urgent or emergency care if necessary. In March 2020, 95% of surveyed dental offices were either entirely closed, or closed and seeing emergency patients only. This also resulted in patients’ lack of prioritization of oral health care, and delay in obtaining care [5]. These changes caused an exacerbation of disparities already present amongst communities already at elevated risk [9]. While several systemic issues exist to prevent marginalized communities from receiving equal access to care, reduced emphasis on the importance of dental care during the COVID-19 pandemic resulted in deeper oral health disparities within the population [9]. Additionally, with shrinking of public health insurance benefits like the Medicaid in several states, suspension of preventive dental care programs such as free clinics and school sealant programs in areas with a shortage of dental providers, thousands if not millions of adults and children were left without the care they may otherwise not have access to [10]. In 2020, Northridge et al. reported that “in response to fiscal challenges, many states have reduced or eliminated Medicaid dental coverage over the past decade, with a concurrent 10% decline in oral health care utilization among low-income adults” [9]. Regarding at-risk populations who have dental benefits under Medicaid, they further report that there is often “difficulty finding Medicaid-contracted dental providers, because only 20% of dentists nationwide accept Medicaid [10].”

Literature notes, in respiratory conditions such as COVID-19, potential mechanisms of pathogenesis include aspiration of oral pathogens into the lungs, alteration of respiratory tract mucosal surfaces to favor adhesion of pathogens, and secretion of hydrolytic enzymes from pathogens that inhibit the innate immune response within the respiratory tract [11]. Additionally, several studies have demonstrated a connection between poor oral hygiene and conditions such as pneumonia, or good oral hygiene and reduced incidence of respiratory disease [11]. There is also evidence that the virus may reside and replicate within periodontal pockets [11]. The symbiotic relationships between microorganisms in the oral cavity are disrupted by poor oral hygiene and periodontal disease. Bacteria in a disturbed biofilm further stimulates cytokine release, in addition to those triggered by the condition of periodontitis alone. These cytokines, upon aspiration, may induce infection and inflammation in the lungs. In early stages of infection, throat is a key area of replication of the virus [11]. It has been shown that within the first week of infection, patients infected with SARS-CoV-2 had elevated concentrations of viral RNA in oropharyngeal swabs, indicating active replication in the region [11]. Severity of infection with SARS-CoV-2 appears to be amplified as a result of comorbidities such as diabetes, hypertension, and cardiovascular disease. These comorbid conditions also have a connection with periodontal disease [6, 7, 11]. While a direct causal relationship cannot be established, it is possible that periodontal disease can intensify the severity of a COVID-19 infection through mechanisms such as enhancing inflammatory responses, causing microbial dysbiosis, and immune system overstimulation [11, 12]. A study performed by Larvin et al. investigated a potential impact of periodontal disease on hospital admission and mortality associated with COVID-19 [13]. While the study could not conclusively link periodontal disease with an increased risk of infection, it was found that within the sample population of patients infected with COVID-19, there was significantly higher mortality amongst participants with periodontal disease [13].

The primary objective of this study is to assess the disproportionate impact of the COVID-19 pandemic (long haul COVID) on populations from lower socioeconomic status in the state of Indiana. The study also aims to assess the association of self-reported periodontal disease and COVID-19 disease course and severity. Considering all the above factors, it is hypothesized that the COVID-19 pandemic did in fact heighten oral health disparities.

## Materials and methods

The Indiana Clinical and Translational Sciences Institute (CTSI) was contacted to identify a sample population with the inclusion criteria of 1) resident of the state of Indiana and 2) positive diagnosis of SARS-CoV-2 in the past year. The sociodemographic distribution (age, gender, race/ethnicity) and information about the social determinants of health for this cohort (income, zip code/neighborhoods and education levels) were also requested. Information about the research study was shared with the cohort, and participants were given a study information sheet detailing its purpose, procedures, risks, and benefits. After reviewing the information sheet, participants were asked to indicate their consent electronically before proceeding to the survey. This method ensured that consent was documented and stored securely within the REDCap platform. This study was IRB exempt. The IRB number associated with the research is #15239.

Upon completion of this portion of the project, a questionnaire was sent to the cohort via the CTSI inquiring whether they are experiencing any symptoms of “long haul COVID.” The survey tool was developed from validated questionnaire available from literature [14–16]. Questions relevant to long haul COVID included inquiring about symptoms such as cough, muscle pain, and loss of taste/smell after the resolution of the infection. The survey tool also inquired about the course of COVID-19 disease progression (mild/moderate/severe ‐ requiring hospitalization), symptoms of gum disease, oral hygiene habits, and presence of co-morbid chronic conditions (such as diabetes and cardiovascular issues). Example questions are, “*How do you feel about your sense of taste compared with the status prior to COVID-19*?” to determine if a participant is still experiencing symptoms after a positive COVID diagnosis, and “*Have you visited an oral healthcare center (dental clinic) for the following in the past 12 months for treatment for gum disease such as scaling and deep cleaning*?” to determine if the participant has a history of periodontal disease and received any treatment for it previously. The survey can be found in S1 Appendix, and was administered via REDCap, a secure virtual platform. Once completed surveys were returned, the data was consolidated and analyzed to best align it with the research question.

Associations of patient characteristics and self-reported periodontal-related survey items with COVID-related survey items were evaluated using chi-square tests, considering the ordinal response categories when appropriate. Spearman correlation coefficients also were calculated when both sets of response categories were ordinal. A two-sided 5% significance level was used for all tests. Analyses were performed using SAS version 9.4 (SAS Institute, Inc., Cary, NC, USA).

## Results

The results of the study underscore the intricate link between periodontal disease, socioeconomic factors, and long-term effects of COVID-19. The percentages represent the proportion of respondents within each category who reported a specific outcome, while ’n’ values denote the actual number of individuals within those percentages. When reporting multiple categories, the proportion was calculated out of our total sample (209) respondents.

The comprehensive analysis included a cohort of 209 individuals from Indiana, who in the past year, tested positive for COVID-19. The demographic data, from “**Table 1**”, reveal a predominant representation of females (n = 154; 74%), while male respondents accounted for a smaller percentage (n = 55; 26%). The age distribution showcased a relatively balanced spread across the different age brackets, with a marginally higher prevalence in the 30–39 years (n = 48; 23%) and 50–64 years (n = 52; 25%) age groups. In terms of racial demographics, most participants were Whites (n = 18; 87%), followed by other racial categories comprising remaining 13% of the participant population. The educational background of the participants was notably high, with most of the participants (n = 122; 59%) holding a college or a postgraduate degree (n = 73; 35%). Employment status predominantly included employed for wages (n = 135; 65%) individuals. The income distribution indicated that a significant portion of the cohort, (n = 93; 44%), had an annual income of $75,000 or more. COVID vaccine uptake among the surveyed individuals shows that the majority (n = 131; 63%), reported having received the initial vaccine series plus one booster shot.

**Table 1:** Respondent demographics.

	Respondent Demographics	
Survey Item	Response	N (%)
**Sex**	Female	154 (74%)
	Male	55 (26%)
**Gender**	Woman	152 (73%)
	Man	54 (26%)
	Other	2 (1%)
	Prefer not to answer	1 (<1%)
**Age**	<30	37 (18%)
	30–39	48 (23%)
	40–49	41 (19%)
	50–64	52 (25%)
	> = 65	31 (15%)
**Race**	White	181 (87%)
	African Americans	14 (6%)
	Asian	6 (3%)
	Hispanic/Latino/African American	7 (3%)
	Pacific Islander, White	1 (<1%)
**Education**	Grade 12 or GED (high school graduate)	14 (6%)
	College graduate (Some college/bachelor’s degree)	122 (59%)
	Post-graduate (master’s degree or higher)	73 (35%)
**Employment**	Employed for wages	140 (67%)
	Unemployed	69 (32%)
**Income**	Less than $25,000	25 (12%)
	Less than $50,000	43 (21%)
	Less than $75,000	29 (14%)
	$75,000 or more	93 (44%)
	Prefer not to answer	19 (9%)
**COVID vaccine**	no vaccine	11 (5%)
	1 shot	8 (4%)
	2 shots	30 (14%)
	vaccine + 1 booster	131 (63%)
	vaccine + 2 boosters	29 (14%)

“**Table 2**” reveals a significant correlation between past COVID-19 hospitalization and oral health practices; Past COVID-19 hospitalization was associated with more frequent dental floss use (p = 0.049), more frequent rinsing mouth (p = 0.041), and treatment of gum diseases within the past 12 months (p<0.001). It must be noted, our sample only had 11 participants (5%) who had reported hospitalization due to COVID-19. Among participants who reported a history of hospitalization, a higher percentage used dental floss once or more in a day (n = 9; 4%). Similarly, most participants with past hospitalization rinsed their mouth once or more in a day (n = 10; 5%). And lastly, many participants who reported hospitalization, visited a clinic in the past 12 months for the treatment of gum disease (n = 7; 22%). There was no statistically significant association between self-reported periodontal disease factors (detected loss of bone around teeth, permanent teeth lost due to gum disease, bleeding gums and previous diagnosis of gum disease), socio-demographic factors, or oral hygiene variables, with past COVID-19 hospitalization.

**Table 2:** Association of past hospitalization due to COVID-19 with oral health and hygiene.

	Past Hospitalization			
*Survey Item*	Response	Yes	No	p-value
**sex**	Female	6 (4%)	148 (96%)	0.140
	Male	5 (9%)	50 (91%)	
**age**	<30	1 (3%)	36 (97%)	0.209
	30–39	1 (2%)	47 (98%)	
	40–49	3 (7%)	38 (93%)	
	50–64	4 (8%)	48 (92%)	
	> = 65	2 (6%)	29 (94%)	
**race**	White	8 (4%)	173 (96%)	0.166
	Non-White	3 (11%)	25 (89%)	
**education**	Grade 12 or GED	1 (7%)	13 (93%)	0.271
	College 1–3 years	4 (10%)	37 (90%)	
	College graduate	3 (4%)	78 (96%)	
	Post-graduate	3 (4%)	70 (96%)	
**employment**	Employed	7 (5%)	133 (95%)	0.574
	Not Working		16 (100%)	
	retired	3 (9%)	29 (91%)	
	student	1 (5%)	20 (95%)	
**income**	Less than $25,000	2 (8%)	23 (92%)	0.666
	Less than $50,000	2 (5%)	41 (95%)	
	Less than $75,000	2 (7%)	27 (93%)	
	$75,000 or more	3 (3%)	90 (97%)	
	Prefer not to answer	2 (11%)	17 (89%)	
**health status**	poor	1 (13%)	7 (88%)	0.126
	fair		15 (100%)	
	good	8 (10%)	71 (90%)	
	very good	1 (1%)	71 (99%)	
	excellent	1 (3%)	34 (97%)	
**bleeding gums**	All of the time		1 (100%)	0.564
	Most of the time	1 (11%)	8 (89%)	
	Some of the time		26 (100%)	
	A little of the time	1 (4%)	24 (96%)	
	None of the time	9 (6%)	139 (94%)	
**mobile or loose tooth**	All of the time		1 (100%)	0.213
	Most of the time	1 (50%)	1 (50%)	
	Some of the time		7 (100%)	
	A little of the time	1 (11%)	8 (89%)	
	None of the time	9 (5%)	181 (95%)	
**you might have gum disease**	Yes	1 (3%)	39 (98%)	0.470
	No	8 (5%)	141 (95%)	
	Don’t know	2 (10%)	18 (90%)	
**loss of teeth**	All of the time		3 (100%)	0.980
	Most of the time		1 (100%)	
	Some of the time		9 (100%)	
	A little of the time	2 (50%)	2 (50%)	
	None of the time	9 (5%)	183 (95%)	
**toothache**	All of the time		3 (100%)	0.520
	Most of the time	1 (17%)	5 (83%)	
	Some of the time		15 (100%)	
	A little of the time	4 (10%)	35 (90%)	
	None of the time	6 (4%)	140 (96%)	
**oral checkup**	> 5 years		5 (100%)	0.372
	> 2 years but < 5 years		16 (100%)	
	> 1 year but < 2 years	2 (6%)	29 (94%)	
	< 1 year	9 (6%)	148 (94%)	
**oral health rating**	poor		6 (100%)	0.963
	fair	2 (5%)	35 (95%)	
	good	6 (8%)	66 (92%)	
	very good		60 (100%)	
	excellent	3 (9%)	31 (91%)	
**brush teeth**	Once a day	4 (6%)	63 (94%)	0.206
	Twice a day	3 (2%)	119 (98%)	
	More than twice a day	4 (20%)	16 (80%)	
**use dental floss**	Never	2 (4%)	46 (96%)	0.049*
	Once a day	6 (4%)	130 (96%)	
	Twice a day		13 (100%)	
	More than twice a day	3 (25%)	9 (75%)	
**rinse mouth**	Never	1 (3%)	34 (97%)	0.041*
	Once a day	3 (4%)	70 (96%)	
	Twice a day	1 (2%)	61 (98%)	
	More than twice a day	6 (15%)	33 (85%)	
**oral cleaning**	Brush and toothpaste	3 (5%)	53 (95%)	0.843
	Brush, toothpaste and floss	8 (5%)	139 (95%)	
	other		6 (100%)	
**detected loss of bone around teeth**	Yes	1 (3%)	32 (97%)	0.718
	No	9 (5%)	156 (95%)	
	Don’t know	1 (9%)	10 (91%)	
**visited clinic past 12 months treatment of gum diseases**	Yes	7 (22%)	25 (78%)	< .001*
	No	4 (2%)	164 (98%)	
	Don’t know		9 (100%)	
**permanent teeth lost gum disease**	Yes	1 (7%)	14 (93%)	0.427
	No	9 (5%)	179 (95%)	
	Don’t know	1 (17%)	5 (83%)	
**COVID vaccine**	no vaccine	2 (18%)	9 (82%)	0.259*
	1 shot	1 (13%)	7 (88%)	
	2 shots	1 (3%)	29 (97%)	
	vaccine + 1 booster	4 (3%)	127 (97%)	
	vaccine + 2 boosters	3 (10%)	26 (90%)	

“**Table 3**” shows the association of sense of smell with socio-demographic and oral factors. Reports of a worsened sense of smell were associated with lower education levels (p = 0.121). A greater proportion of individuals with Grade 12 or GED level of education reported worsened sense of smell (n = 6, 43%) than individuals with higher levels of education such as post-graduate (n = 19, 26%). In addition, lowered sense of smell was significantly more common amongst participants who were unemployed or disabled (p = 0.008). The survey results indicated that a greater percentage of unemployed individuals (n = 12; 75%) had a worsened sense of smell post COVID-19 infection compared to their employed counterparts. Furthermore, there was a significant association observed between the participants having a worse general health status (p<0.001), bleeding gums (p = 0.031), history of toothaches (p = 0.000) and a lower oral health rating (p = 0.002). Among the respondents, a significant number of those who reported a total loss of or sense of smell worse than before (n = 18; 9%), also reported having fair to poor general health status. Among the same group, a significant number also reported having bleeding gums (n = 33, 16%) and a history of toothaches (n = 38, 18%). A majority of participants with a self-assessed oral health rating of ’poor,’ (n = 4; 67%) reported a worsened sense of smell post-COVID when compared with those who had an oral health rating of ‘very good’ (n = 16; 27%) or ‘excellent’ (n = 8; 24%). Lastly, a significant association was also noted between COVID-19 vaccination status and sense of smell (p = 0.011). A greater percentage of participants reported a worsened sense of smell if they had not received the vaccine (n = 7, 64%). A significantly lower percentage of participants reported worsened sense of smell if they had received a booster (n = 41, 31% and n = 7, 24%).

**Table 3:** Association of altered sense of smell with demographic and oral health factors.

		Sense of smell compared with pre-COVID					
*Survey Item*	Response	Total loss	Worse than before	Same as before	Better than before	p-value	Spearman Correlation
**sex**	Female	2 (1%)	57 (37%)	92 (60%)	3 (2%)	0.224	
	Male		17 (31%)	36 (65%)	2 (4%)		
**age**	<30		9 (24%)	27 (73%)	1 (3%)	0.090	-0.10
	30–39		16 (33%)	30 (63%)	2 (4%)		
	40–49		20 (49%)	21 (51%)			
	50–64		19 (37%)	31 (60%)	2 (4%)		
	> = 65	2 (6%)	10 (32%)	19 (61%)			
**race**	White	2 (1%)	62 (34%)	113 (62%)	4 (2%)	0.649	
	Non-White		12 (43%)	15 (54%)	1 (4%)		
**education**	Grade 12 or GED		6 (43%)	8 (57%)		0.021*	0.19
	College 1–3 years		24 (59%)	16 (39%)	1 (2%)		
	College graduate	1 (1%)	26 (32%)	50 (62%)	4 (5%)		
	Post-graduate	1 (1%)	18 (25%)	54 (74%)			
**employment**	Employed	2 (1%)	47 (34%)	87 (62%)	4 (3%)	0.008*	
	Not Working		12 (75%)	4 (25%)			
	Retired		11 (34%)	21 (66%)			
	Student		4 (19%)	16 (76%)	1 (5%)		
**income**	Less than $25,000		7 (28%)	17 (68%)	1 (4%)	0.158	0.05
	Less than $50,000	1 (2%)	18 (42%)	21 (49%)	3 (7%)		
	Less than $75,000		14 (48%)	15 (52%)			
	$75,000 or more		27 (29%)	65 (70%)	1 (1%)		
	Prefer not to answer	1 (5%)	8 (42%)	10 (53%)			
**health status**	poor		6 (75%)	2 (25%)		0.000*	0.31
	fair		12 (80%)	3 (20%)			
	good	1 (1%)	33 (42%)	43 (54%)	2 (3%)		
	very good		17 (24%)	52 (72%)	3 (4%)		
	excellent	1 (3%)	6 (17%)	28 (80%)			
**bleeding gums**	All of the time		1 (100%)			0.031*	0.19
	Most of the time		6 (67%)	1 (11%)	2 (22%)		
	Some of the time		12 (46%)	14 (54%)			
	A little of the time		14 (56%)	10 (40%)	1 (4%)		
	None of the time	2 (1%)	41 (28%)	103 (70%)	2 (1%)		
**mobile or loose tooth**	All of the time		1 (100%)			0.142	0.09
	Most of the time		1 (50%)	1 (50%)			
	Some of the time		5 (71%)	1 (14%)	1 (14%)		
	A little of the time		3 (33%)	6 (67%)			
	None of the time	2 (1%)	64 (34%)	120 (63%)	4 (2%)		
**might have gum disease**	Yes		21 (53%)	17 (43%)	2 (5%)	0.076	
	No	1 (1%)	44 (30%)	102 (68%)	2 (1%)		
	Don’t know	1 (5%)	9 (45%)	9 (45%)	1 (5%)		
**loss of teeth**	All of the time		3 (100%)			0.051	0.15
	Most of the time			1 (100%)			
	Some of the time		5 (56%)	3 (33%)	1 (11%)		
	A little of the time		3 (75%)	1 (25%)			
	None of the time	2 (1%)	63 (33%)	123 (64%)	4 (2%)		
**toothache**	All of the time		2 (67%)	1 (33%)		0.000*	0.31
	Most of the time		4 (67%)	1 (17%)	1 (17%)		
	Some of the time		11 (73%)	4 (27%)			
	A little of the time		21 (54%)	17 (44%)	1 (3%)		
	None of the time	2 (1%)	36 (25%)	105 (72%)	3 (2%)		
**oral checkup**	> 5 years		4 (80%)	1 (20%)		0.512	0.04
	> 2 years but < 5 years	1 (6%)	2 (13%)	12 (75%)	1 (6%)		
	> 1 year but < 2 years		14 (45%)	16 (52%)	1 (3%)		
	< 1 year	1 (1%)	54 (34%)	99 (63%)	3 (2%)		
**oral health rating**	poor		4 (67%)	2 (33%)		0.002*	0.22
	fair	1 (3%)	20 (54%)	15 (41%)	1 (3%)		
	good		26 (36%)	44 (61%)	2 (3%)		
	very good	1 (2%)	16 (27%)	42 (70%)	1 (2%)		
	excellent		8 (24%)	25 (74%)	1 (3%)		
**brush teeth**	Once a day	1 (1%)	21 (31%)	43 (64%)	2 (3%)	0.255	-0.07
	Twice a day		45 (37%)	74 (61%)	3 (2%)		
	More than twice a day	1 (5%)	8 (40%)	11 (55%)			
**use dental floss**	Never		15 (31%)	33 (69%)		0.056	-0.11
	Once a day	2 (1%)	45 (33%)	84 (62%)	5 (4%)		
	Twice a day		6 (46%)	7 (54%)			
	More than twice a day		8 (67%)	4 (33%)			
**rinse mouth**	Never		11 (31%)	23 (66%)	1 (3%)	0.063	-0.13
	Once a day	1 (1%)	20 (27%)	50 (68%)	2 (3%)		
	Twice a day		26 (42%)	35 (56%)	1 (2%)		
	More than twice a day	1 (3%)	17 (44%)	20 (51%)	1 (3%)		
**oral cleaning**	Brush and toothpaste	2 (4%)	21 (38%)	32 (57%)	1 (2%)	0.346	
	Brush, toothpaste and floss		52 (35%)	91 (62%)	4 (3%)		
	other		1 (17%)	5 (83%)			
**detected loss of bone around teeth**	Yes	1 (3%)	17 (52%)	14 (42%)	1 (3%)	0.056	
	No	1 (1%)	55 (33%)	105 (64%)	4 (2%)		
	Don’t know		2 (18%)	9 (82%)			
**visited clinic past 12 months treatment of gum diseases**	Yes	1 (3%)	12 (38%)	18 (56%)	1 (3%)	0.353	
	No	1 (1%)	60 (36%)	104 (62%)	3 (2%)		
	Don’t know		2 (22%)	6 (67%)	1 (11%)		
**permanent teeth lost gum disease**	Yes	1 (7%)	5 (33%)	8 (53%)	1 (7%)	0.077	
	No	1 (1%)	64 (34%)	119 (63%)	4 (2%)		
	Don’t know		5 (83%)	1 (17%)			
**COVID vaccine**	no vaccine		7 (64%)	4 (36%)		0.011*	0.19
	1 shot		3 (38%)	5 (63%)			
	2 shots		16 (53%)	13 (43%)	1 (3%)		
	vaccine + 1 booster	1 (1%)	41 (31%)	88 (67%)	1 (1%)		
	vaccine + 2 boosters	1 (3%)	7 (24%)	18 (62%)	3 (10%)		

“**Table 4**” shows association between sense of taste post COVID-19 and socio-demographic and oral factors. Lower sense of taste ratings were associated with older age (p = 0.018). Participants within the age group of 40–49 years reported a worsened sense of taste at a higher frequency than others (n = 19; 46%); followed by the >65 years age group (n = 12; 39%). The least affected group was those under the age of thirty (n = 7; 19%). Lower sense of taste was also associated with worse overall health status (p<0.001), mobile or loose teeth (p = 0.010), tooth loss (p = 0.013), bleeding gums (p<0.001), possibility of gum disease (p<0.001), permanent teeth lost due to gum disease (p = 0.006), toothache (p<0.001), and lower oral health ratings (p = 0.001). Among the respondents, a significant number of those who reported a total loss of or sense of taste worse than before (n = 18; 9%), also reported having fair to poor general health status. Among the same group, a significant number also reported having mobile or loose teeth (n = 12, 6%) a history of tooth loss (n = 11, 5%), bleeding gums (n = 35, 17%), possibility of gum disease (n = 26, 12%), permanent tooth loss due to gum disease (n = 10, 5%), and a history of toothaches (n = 34, 16%). Most participants with a self-assessed oral health rating of ’poor,’ (n = 4; 67%) reported a worsened sense of taste post-COVID when compared with those who had an oral health rating of ‘very good’ (n = 14; 23%) or ‘excellent’ (n = 10; 29%). And lastly, a significant association was noted between COVID-19 vaccine and sense of taste (p = 0.001). A greater percentage of participants reported a worsened sense of taste post COVID-19 if they had not received the vaccine (n = 8, 73%). A significantly lower percentage of participants reported worsened sense of taste if they had received the vaccine and booster(s) (n = 35, 27% and n = 9, 31%).

**Table 4:** Association of altered sense of taste with demographic and oral health factors.

		Sense of taste compared with pre-COVID					
*Survey Item*	Response	Total loss	Worse than before	Same as before	Better than before	p-value	Spearman Correlation
**sex**	Female	3 (2%)	52 (34%)	98 (64%)	1 (1%)	0.476	
	Male		20 (36%)	32 (58%)	3 (5%)		
**age**	<30		7 (19%)	30 (81%)		0.018*	-0.16
	30–39	1 (2%)	14 (29%)	31 (65%)	2 (4%)		
	40–49		19 (46%)	22 (54%)			
	50–64		20 (38%)	30 (58%)	2 (4%)		
	> = 65	2 (6%)	12 (39%)	17 (55%)			
**race**	White	3 (2%)	63 (35%)	113 (62%)	2 (1%)	0.278	
	Non-White		9 (32%)	17 (61%)	2 (7%)		
**education**	Grade 12 or GED		6 (43%)	8 (57%)		0.059	0.15
	College 1–3 years	1 (2%)	22 (54%)	16 (39%)	2 (5%)		
	College graduate	1 (1%)	24 (30%)	54 (67%)	2 (2%)		
	Post-graduate	1 (1%)	20 (27%)	52 (71%)			
**employment**	Employed	3 (2%)	43 (31%)	91 (65%)	3 (2%)	0.075	
	Not Working		10 (63%)	6 (38%)			
	Retired		15 (47%)	16 (50%)	1 (3%)		
	Student		4 (19%)	17 (81%)			
**income**	Less than $25,000		8 (32%)	15 (60%)	2 (8%)	0.087	0.09
	Less than $50,000	2 (5%)	17 (40%)	24 (56%)			
	Less than $75,000		15 (52%)	14 (48%)			
	$75,000 or more		27 (29%)	65 (70%)	1 (1%)		
	Prefer not to answer	1 (5%)	5 (26%)	12 (63%)	1 (5%)		
**health status**	poor		6 (75%)	2 (25%)		< .001*	0.25
	fair		12 (80%)	3 (20%)			
	good	1 (1%)	27 (34%)	49 (62%)	2 (3%)		
	very good	1 (1%)	22 (31%)	47 (65%)	2 (3%)		
	excellent	1 (3%)	5 (14%)	29 (83%)			
**bleeding gums**	All of the time		1 (100%)			< .001*	0.28
	Most of the time	1 (11%)	6 (67%)	2 (22%)			
	Some of the time		15 (58%)	11 (42%)			
	A little of the time		12 (48%)	11 (44%)	2 (8%)		
	None of the time	2 (1%)	38 (26%)	106 (72%)	2 (1%)		
**mobile or loose tooth**	All of the time		1 (100%)			0.010*	0.19
	Most of the time		1 (50%)	1 (50%)			
	Some of the time	1 (14%)	3 (43%)	3 (43%)			
	A little of the time		6 (67%)	3 (33%)			
	None of the time	2 (1%)	61 (32%)	123 (65%)	4 (2%)		
**you might have gum disease**	Yes	1 (3%)	25 (63%)	14 (35%)		< .001*	
	No	1 (1%)	41 (28%)	104 (70%)	3 (2%)		
	Don’t know	1 (5%)	6 (30%)	12 (60%)	1 (5%)		
**loss of teeth**	All of the time		3 (100%)			0.013*	0.17
	Most of the time			1 (100%)			
	Some of the time	1 (11%)	4 (44%)	4 (44%)			
	A little of the time		3 (75%)		1 (25%)		
	None of the time	2 (1%)	62 (32%)	125 (65%)	3 (2%)		
**toothache**	All of the time		2 (67%)	1 (33%)		< .001*	0.26
	Most of the time		5 (83%)	1 (17%)			
	Some of the time		9 (60%)	6 (40%)			
	A little of the time	1 (3%)	17 (44%)	20 (51%)	1 (3%)		
	None of the time	2 (1%)	39 (27%)	102 (70%)	3 (2%)		
**oral checkup**	> 5 years		4 (80%)	1 (20%)		0.266	0.05
	> 2 years but < 5 years	2 (13%)	1 (6%)	13 (81%)			
	> 1 year but < 2 years		14 (45%)	15 (48%)	2 (6%)		
	< 1 year	1 (1%)	53 (34%)	101 (64%)	2 (1%)		
**oral health rating**	poor		4 (67%)	2 (33%)		0.001*	0.23
	fair	2 (5%)	20 (54%)	15 (41%)			
	good		24 (33%)	46 (64%)	2 (3%)		
	very good	1 (2%)	14 (23%)	44 (73%)	1 (2%)		
	excellent		10 (29%)	23 (68%)	1 (3%)		
**brush teeth**	Once a day	2 (3%)	16 (24%)	49 (73%)		0.163	-0.12
	Twice a day		47 (39%)	72 (59%)	3 (2%)		
	More than twice a day	1 (5%)	9 (45%)	9 (45%)	1 (5%)		
**use dental floss**	Never		13 (27%)	35 (73%)		0.081	-0.12
	Once a day	3 (2%)	46 (34%)	84 (62%)	3 (2%)		
	Twice a day		6 (46%)	6 (46%)	1 (8%)		
	More than twice a day		7 (58%)	5 (42%)			
**rinse mouth**	Never		9 (26%)	26 (74%)		0.203	-0.10
	Once a day	2 (3%)	22 (30%)	49 (67%)			
	Twice a day		24 (39%)	36 (58%)	2 (3%)		
	More than twice a day	1 (3%)	17 (44%)	19 (49%)	2 (5%)		
**oral cleaning**	Brush and toothpaste	2 (4%)	17 (30%)	36 (64%)	1 (2%)	0.995	
	Brush, toothpaste and floss	1 (1%)	53 (36%)	90 (61%)	3 (2%)		
	other		2 (33%)	4 (67%)			
**detected loss of bone around teeth**	Yes	1 (3%)	16 (48%)	14 (42%)	2 (6%)	0.304	
	No	2 (1%)	53 (32%)	108 (65%)	2 (1%)		
	Don’t know		3 (27%)	8 (73%)			
**visited clinic past 12 months treatment of gum diseases**	Yes	1 (3%)	14 (44%)	16 (50%)	1 (3%)	0.356	
	No	2 (1%)	55 (33%)	109 (65%)	2 (1%)		
	Don’t know		3 (33%)	5 (56%)	1 (11%)		
**permanent teeth lost gum disease**	Yes	2 (13%)	8 (53%)	4 (27%)	1 (7%)	0.006*	
	No	1 (1%)	60 (32%)	124 (66%)	3 (2%)		
	Don’t know		4 (67%)	2 (33%)			
**COVID vaccine**	no vaccine		8 (73%)	3 (27%)		0.001*	0.23
	1 shot		4 (50%)	4 (50%)			
	2 shots	1 (3%)	16 (53%)	13 (43%)			
	vaccine + 1 booster	1 (1%)	35 (27%)	94 (72%)	1 (1%)		
	vaccine + 2 boosters	1 (3%)	9 (31%)	16 (55%)	3 (10%)		

## Discussion

The study’s primary focus was to investigate the impact of COVID-19 on lower socioeconomic populations and its association with self-reported periodontal disease. It has been demonstrated that the COVID-19 pandemic and all of the changes that came with it–dental offices closing temporarily, free health care programs being suspended, and patient lack of pursuit of dental treatment for a variety of reasons–largely stood in the way of patients obtaining dental care necessary for their well-being. As a result, the oral health status of marginalized populations deteriorated. Literature also notes the association between income loss during the COVID-19 pandemic and unmet dental care for children, emphasizing the financial barriers to dental care during the pandemic [17]. Oral health is connected to overall health, and a worsening condition of the oral cavity, potentially combined with comorbid conditions, can impact the course of an individual’s COVID-19 infection, potentially increasing the severity and increasing the probability for symptoms remaining long term.

While some results were statistically significant, with only eleven hospitalizations (5%) observed in the sample, some frequencies reported were higher in opposing categories. It is important to note that while these statistically significant associations between variables provide valuable insights, they may not necessarily indicate direct causal relationships due to the limitations of a small sample size and data scope. Therefore, it is also important to emphasize that the study’s results are not entirely generalizable.

When considering demographic factors, age was the sole variable that displayed a significant association with changes in gustatory function following a COVID-19 infection. This is consistent with a recent study by Perlis et al. which found that long haul COVID was associated with older age and female sex [18]. Per our study results, for changes in olfactory function, education and employment were the only socio-demographic factors that showed statistical significance in relation to changes in sense of smell compared to pre-COVID. Individuals with lower levels of education seemed to report a worsened sense of smell, along with individuals that are unemployed as opposed to those who are not. These results align with our study’s overarching theme of addressing disparities in oral health, supporting the notion that socioeconomic factors impacted access to dental care during the COVID-19 pandemic. This may be contrasting with a cross-sectional study by Mahmoodi et al. found that individuals with higher education levels and underlying comorbid conditions were at greater risk for having symptoms of long haul COVID [19]. Despite this contrast between the two studies, one portion of Mahmoodi et al.’s findings does align with results found in our data; individuals with lower general health status were more likely to report having symptoms of long haul COVID such as worsened sense of smell or taste.

Periodontal disease factors such as bleeding gums, previous diagnosis of gum disease, and permanent teeth lost due to gum disease exhibited significant association with a worsened sense of taste post COVID-19. As for worsened sense of smell post COVID-19, only bleeding gums of all the self-reported gum disease symptoms was found to be associated. Similar to our results, a case control study performed by Sari et al. concluded that poor periodontal health and higher incidence of periodontitis was observed in populations that was recently infected by COVID-19 [20]. While our study cannot attribute a direct association between self-reported symptoms of periodontal disease and history of hospitalization, a case control study by Marouf et al. suggests periodontal disease was associated with COVID-19 complications including admission to the hospital, need for assisted ventilation, and even death [21]. This is due to a possible mechanism of increased local and systemic inflammatory responses [20, 21]. Lastly, a study by Costa et al. determined that there was a positive association between oral health conditions such as periodontitis and severe COVID-19 outcomes such as hospitalization [22]. This study focused on immediate consequences within a clinical setting, while our study uniquely explores post-COVID-19 sensory changes in a broader demographic context [22]. This comparison highlights the complementary nature of the two studies, offering a more comprehensive understanding of the diverse impacts of COVID-19 on oral health outcomes.

Interestingly, despite the expected potential benefits of oral hygiene practices, our study revealed that oral hygiene practices such as brushing teeth, using dental floss, and mouth rinsing were not significantly associated with reports of altered sense of taste or smell. In contrast to this, a study by Catton et al. indicates a link between flossing and rapid taste recovery, suggesting that oral hygiene practices may actually reduce COVID-19 viral entry and dissemination [23]. As mentioned previously, with only eleven hospitalizations (5%) observed in the sample, some frequencies reported were higher in opposing categories. Our results in this category may not indicate direct causal relationships as a result of the limitations of a small sample size and data scope.

With regards to vaccination status, individuals who had not been vaccinated for COVID-19 generally reported worsened levels of both sense of smell and taste. Additionally, those who had not received a COVID-19 booster also reported worsened levels of sense of smell and taste. A recent study by Perlis et al. found that the risk of long-term COVID was reduced in populations that received the primary vaccination series [18]. This complements results from other studies–vaccination generally results in a less severe disease course. And a less severe disease course is associated with a lower incidence of long haul COVID symptoms, such as worsened smell or taste. Research suggests that vaccination may alleviate certain long-COVID symptoms. This potential improvement could be due to an enhanced immune response triggered by the vaccine. Conversely, per a recent study, those who received at least one dose of a COVID-19 vaccine were more likely to report prolonged long-COVID symptoms more than a year after infection [24].

A study by Strain et al., noted that the Moderna mRNA-1273 vaccine showed the most significant improvement in long COVID symptoms (66% improvement vs. 12% deterioration), followed by Pfizer-BioNTech BNT162b2 (56% improvement vs. 18% deterioration) and Oxford-AstraZeneca ChAdOx1 nCoV-19 (58% improvement vs. 19% deterioration) [25]. In contrast, some studies indicated that the overall impact of vaccine type on symptom changes was minimal, with no significant differences reported between the types of vaccines [26–28]. However, many studies have had small sample sizes, lacked diverse representation, and didn’t account for pre-pandemic symptoms or include a never-infected comparison group, which could show similar nonspecific symptoms [29]. Despite these mixed findings, vaccines are known to significantly reduce the severity of COVID-19, including the risk of hospitalization and death [30]. The observed association between vaccination and prolonged symptoms does not diminish these protective benefits but underscores the need for further research. Specifically, future studies should explore how different vaccines might influence long-COVID persistence and impact long-term oral health outcomes. Understanding these interactions, especially in relation to pre-existing conditions like periodontal disease, could provide valuable insights into post-COVID recovery.

### Strengths and weaknesses

Our study provides valuable insights into the progression and long-term effects of COVID-19, especially in relation to oral health. This approach is complemented by our attention to diverse demographic and socioeconomic factors, enriching our understanding of the pandemic’s impact across different population groups. These findings shed light on the complex interplay between oral health, socio-demographic factors, and COVID-19 outcomes, highlighting the relevance of oral health in understanding the impact of the pandemic and inform future public health strategies and research directions.

Conversely, there are several limitations present in our study. The study data was cross sectional, which does not confer causality. The reliance on self-reported data for periodontal disease diagnosis is a limitation, as it lacks the clinical validation that a comprehensive periodontal examination, including radiographs and clinical assessments, would provide. In addition, self-reported data is often masked by recall bias which again is a big limitation. The small sample size, particularly in subgroups such as those hospitalized due to COVID-19, limits the generalizability of our results. This is evident in the inverse frequency of certain outcomes, such as more hospitalizations among participants who followed oral hygiene practices more religiously, which may be attributed to the small sample size and may not accurately reflect a larger trend. The survey did not collect specific data on the types of COVID-19 vaccines received by participants, which may limit the understanding of the impact of different vaccine types on long-term COVID-19 symptoms. Additionally, because our study population was geographically limited to Indiana, our results may not be generalizable.

### Future implications

Future research should aim at expanding on these findings with a larger, more diverse cohort and multivariable analysis to dissect the complex relationships between sociodemographic variables, oral health and COVID-19 outcomes. Additionally, further investigation into the role of oral health practices and their potential impact on the severity of COVID-19 symptoms could provide valuable insights for public health interventions. Identifying these disparities is crucial for informing policy changes aimed at improving access to oral health care for marginalized groups.

## Conclusion

This study reveals complex links between periodontal disease, socioeconomic factors, and long-term COVID-19 impacts. While associations were identified, limitations such as a small sample size and geographical focus exist. The findings underscore the importance of oral health in the context of the pandemic, suggesting potential connections with COVID-19 outcomes. However, the study also highlights the need for more comprehensive research, emphasizing the intricate nature of these relationships. Addressing oral health disparities, particularly among marginalized groups, is crucial for informed policy changes and improved health outcomes in future public health strategies.

## Supporting information

S1 Appendix Long COVID-19 oral health survey. (PDF)

S1 File (XLSX)
