# Supplementary material for: Association of self-reported periodontal disease and inequities with long haul COVID-19
Source: PLoS One. 2024 Oct 10;19(10):e0311644. doi: 10.1371/journal.pone.0311644 (PMC11469594; doi:10.1371/journal.pone.0311644)
Supplement: S1 Appendix — (PDF) [file pone.0311644.s001.pdf]

Long Covid-19 Oral Health Survey

Researchers at Indiana University are looking for volunteers to take part in a study to learn about persistent oral symptoms in severe acute respiratory system coronavirus-2 (SARS-CoV2) carriers.

This survey asks for your views about your general and oral health.

Please answer each question by choosing just one answer. If you are unsure how to answer a question, please give the best answer you can.

Thank you,

The Long Covid-19 Oral Health Study Team

The following questions are about Covid testing.

What was the approximate date of your first positive Covid test result? ______________________________

Have you tested positive for Covid more than once?

◯ Yes
◯ No

How many times total?:

____

	Yes	No
In the past, were you hospitalized for Covid-19?	◯	◯

Have you received a Covid vaccine? (choose 1 response):

◯ Yes, one injection/shot
◯ Yes, 2 injections/shots
◯ Yes, 2 injections/shot and 1 booster
◯ Yes, 2 injections/shot and 2 boosters
◯ No
◯ Other

Other (please explain): ______________________________

The following questions are about your general activities after testing positive for COVID-19.

	Excellent	Very good	Good	Fair	Poor
In general, would you say your health is:	○	○	○	○	○

In the past year does your health limit you in everyday activities? If so, how much?

	YES, limited a lot	YES, limited a little	NO, not limited at all
Moderate activities such as moving a table, pushing a vacuum cleaner, bowling, or playing golf.	○	○	○
Climbing several flights of stairs.	○	○	○

During the past year after COVID-19 infection, have you had any of the following problems with your work or other regular daily activities as a result of your physical health?

	Yes	No
Accomplished less than you would like.	○	○
Were limited in the kind of work or other activities.	○	○

Have you had any of the following problems with your work or other regular daily activities as a result of any emotional problems (such as feeling depressed or anxious)?

	Yes	No
Accomplished less than you would like.	○	○
Did work or activities less carefully than usual.	○	○

These questions are about general health, how you have been feeling during the past year after COVID-19.

For each question, please give the one answer that comes closest to the way you have been feeling.

Have you experienced any of the following symptoms that appeared post COVID-19? How much of the time during the year have you had...

	All of the time	Most of the time	Some of the time	A little of the time	None of the time
Headache	○	○	○	○	○
Muscle pain or myalgia	○	○	○	○	○
Chest pain	○	○	○	○	○
Joint pain	○	○	○	○	○
Cough	○	○	○	○	○

Any other pain	○	○	○	○	○

Other pain (please specify): ____________________________

	All of the time	Most of the time	Some of the time	A little of the time	None of the time
During the past year after COVID-19, how much of the time has your physical health or emotional problems or oral health interfered with your social activities (like visiting friends, relatives, etc.)?	○	○	○	○	○

These questions are about taste and smell after COVID-19.

For each question, please give the one answer that comes closest to the way you have been feeling.

	Same as before	Worse than before	Better than before	Total loss
How do you feel about your sense of taste compared with the status prior to COVID-19?	○	○	○	○
How do you feel about your sense of smell compared with the status prior to COVID-19?	○	○	○	○

These questions are about your oral health and how you have been feeling during the past year after COVID-19.

For each question, please give the one answer that comes closest to the way you have been feeling.

Have you experienced any of the following symptoms that appeared post COVID-19? How much of the time during the year have you had...

	All of the time	Most of the time	Some of the time	A little of the time	None of the time
Dry mouth	○	○	○	○	○
Bleeding gums	○	○	○	○	○
Toothache	○	○	○	○	○
Mobile or loose tooth	○	○	○	○	○
Loss of tooth/teeth	○	○	○	○	○
Mouth ulcers	○	○	○	○	○
Jaw pain	○	○	○	○	○

Any other pain	◯	◯	◯	◯	◯

Other oral pain (please specify): ____________________

These questions are about your oral health, oral hygiene habits, and and history of gum disease.

When was the last time you got an oral health check-up?

◯ Less than a year
◯ More than a year but less than 2 years
◯ More than 2 years but less than 5 years
◯ More than 5 years
◯ Never have been

Overall, how would you rate the health of your teeth and gums?

◯ Excellent
◯ Very Good
◯ Good
◯ Fair
◯ Poor
◯ Don't know

What do you use for cleaning your teeth?

◯ Floss only
◯ Brush and toothpaste
◯ Brush, toothpaste and floss
◯ Other

Other (please specify):

____

How often do you do the following?

	Never	Once a day	Twice a day	More than twice a day
Brush your teeth	◯	◯	◯	◯
Use dental floss	◯	◯	◯	◯
Clean your tongue	◯	◯	◯	◯
Rinse your mouth	◯	◯	◯	◯

Please choose one answer for each of the following questions:

	Yes	No	Don't Know

Have you visited an oral healthcare center (dental clinic) for the following in the past 12 months for treatment for gum disease such as scaling and deep cleaning?	○	○	○
Not including teeth lost for injury or orthodontics, have you lost any of your permanent teeth due to gum disease?	○	○	○
Gum disease is a common problem with the mouth. People with gum disease might have swollen gums, receding gums, sore or infected gums or loose teeth. Do you think you might have gum disease?	○	○	○
Have you ever been told by a dental professional that you lost bone around your teeth?	○	○	○

Our research study is evaluating saliva for oral and systemic health markers in post COVID-19 population.

	Yes	Maybe	Never
Would you be willing to be contacted for this research study in future that includes an oral examination and saliva sample collection?	○	○	○
